# A new brown rot disease of plum caused by *Mucor xinjiangensis* sp. nov. and screening of its chemical control

**DOI:** 10.3389/fmicb.2024.1458456

**Published:** 2024-09-10

**Authors:** Bo Song, Mubashar Raza, Li-Juan Zhang, Bing-Qiang Xu, Pan Zhang, Xiao-Feng Zhu

**Affiliations:** ^1^Key Laboratory of Integrated Pest Management on Crops in Northwestern Oasis, Ministry of Agriculture and Rural Affairs, Institute of Plant Protection, Xinjiang Academy of Agricultural Sciences, Xinjiang Uyghur Autonomous Region, Urumqi, China; ^2^Xinjiang Key Laboratory of Agricultural Biosafety, Institute of Plant Protection, Xinjiang Academy of Agricultural Sciences, Xinjiang Uyghur Autonomous Regio, Urumqi, China; ^3^Xinjiang Laboratory of Special Environmental Microbiology, Institute of Applied Microbiology, Xinjiang Academy of Agricultural Sciences, Xinjiang Uyghur Autonomous Region, Urumqi, China

**Keywords:** chemical control, new taxon, plant pathogen, *Prunus domestica* (European plum), taxonomy, Xinjiang (China)

## Abstract

A novel species of *Mucor* was identified as the causal agent of a brown rot of *Prunus domestica* (European plum), widely grown in the south of Xinjiang, China. This disease first appears as red spots after the onset of the fruits. With favorable environmental conditions, fruit with infected spots turn brown, sag, expand, wrinkle, and harden, resulting in fruit falling. Fungal species were isolated from infected fruits. A phylogenetic analysis based on internal transcribed spacer (ITS) regions and the large subunit (LSU) of the nuclear ribosomal RNA (rRNA) gene regions strongly supported that these isolates made a distinct evolutionary lineage in *Mucor* (Mucoromycetes, Mucoraceae) that represents a new taxonomic species, herein named as *Mucor xinjiangensis*. Microscopic characters confirmed that these strains were morphologically distinct from known *Mucor* species. The pathogenicity of *M. xinjiangensis* was confirmed by attaching an agar disk containing mycelium on fruits and re-isolation of the pathogen from symptomatic tissues. Later, fourteen fungicides were selected to determine the inhibitory effect on the pathogen. Further, results showed that difenoconazole had the best effect on the pathogen and the strongest toxicity with the smallest half maximal effective concentration (EC_50_) value, followed by a compound fungicide composed of difenoconazole with azoxystrobin, mancozeb, prochloraz with iprodione, pyraclostrobin with tebuconazole, and trifloxystrobin with tebuconazole and ethhylicin. Present study provides the basis for the prevention and control of the novel plum disease and its pathogen.

## Introduction

*Prunus domestica* is a flowering plant species belonging to the family Rosaceae. It is commonly known as European plum, common plum, or prune and produces stone fruits, which are typically called plums (Zhebentyayeva et al., 2019). *Prusnusdomestica* is a rich source of vitamins, minerals, organic acids, and fiber, including anthocyanins, flavonol derivatives, and phenolic acids (Soares et al., 2023). A number of studies have shown that the consumption of plums promotes a wide range of health benefits; prevents a wide variety of diseases, such as cancer, diabetes, and obesity; has anti-inflammatory properties; improves digestive function; and has significant applications in the fields of medicine and food (Silvan et al., 2020; Abraao et al., 2022; Bahrin et al., 2022; Rybak and Wojdyło, 2023).

Over the past few years, plum-growing areas have increased in Xinjiang, China (Wang et al., 2021). The total plum-growing area (in Chinese pinyin: Xinmei) in Kashgar Jiashi County is more than 28,000 ha, yielding an estimated production volume of 85,000 tons. It accounts for 40% of China’s total area of plum production and 60% of China’s total plum output (Fan and Zhang, 2023). Due to the gradual expansion of the plum growth area, coupled with mismanagement and inadequate technology, plums are prone to diseases and pests such as spot disease, brown rot, and food worms, which negatively influence their growth.

Mucor rot is a common postharvest disease of pome and stone fruits such as apples, cherries, nectarines, pears, peaches, plums, and prunes, as well as other commercial berries and citrus fruits. This disease is mainly caused by *Mucor piriformis* and a few other *Mucor* species, such as *Mucor circinelloides* (Saito et al., 2016), *Mucor fragilis* (Abbas et al., 2018; Khan and Javaid, 2022), *Mucor hiemalis* (Saito et al., 2016), *Mucor mucedo* (Eseigbe and Bankole, 1996; Saito et al., 2016), *Mucor racemosus* (Kwon and Hong, 2005; López et al., 2016), and *Mucor strictus* (Suh et al., 2018). However, in cold storage, only *M. piriformis* has been frequently found to cause significant losses (López et al., 2016; Saito et al., 2016). The causal agent of Mucor rot belongs to the genus *Mucor* which is the type of subkingdom Mucoromyceta (Mucoromycota, Mucoromycetes, Mucorales, and Mucoraceae), with conserved type *M. mucedo* (Tedersoo et al., 2018; Turland et al., 2018). Typically, it has fast growth, aerial and luxuriant hyphae, sporangiophores with no branching, and zygospores with opposed suspensors (Schipper, 1978). All over the world, various species of *Mucor* are widely collected from soil and dung (Walther et al., 2013).

In the realm of agricultural practices, combating fungal diseases remains a critical concern, particularly in safeguarding the health and yield of essential agricultural and fruit crops. Despite advancements in various agricultural techniques, chemical control continues to stand out as a fundamental tool in the fight against fungal pathogens (Kettles and Luna, 2019). Currently, *Mucor* species associated with plums were mainly reported from Norway (Børve and Vangdal, 2007), Nigeria (Eseigbe and Bankole, 1996), Pakistan (Hassan et al., 2022), Poland (Tuszyński and Satora, 2003), Saudia Arabia (Gherbawy and Hussein, 2010), South Africa (Kwinda et al., 2015), Turkey (Ghimire et al., 2022), and USA (Hong et al., 2000). Despite the advancements in agricultural practices, the control of *Mucor* species remains a significant concern for farmers and researchers alike. Plant diseases caused by *Mucor* species can be prevented and reduced by various management strategies. There have been some reports to control these diseases caused by fungi, such as *Mucor* species, with fungicides (Koka et al., 2021; Saito et al., 2023), plant extracts (Kinge and Besong, 2021; Jangid and Begum, 2022), and biological control agents (Wallace et al., 2018; Oufensou et al., 2023). The *Mucor* species associated with plums in China have not been reported. In our investigation of plum diseases and their management in China, a total of 37 *Mucor* strains were isolated. Based on the morphological and phylogenetic analysis, these isolates were identified as *Mucor xinjiangensis* sp. nov. A detailed description and illustration are provided for the new species and compared with other closely related taxa. Pathogenicity test confirmed that *M. xinjiangensis* causative agent of brown rot of plum. Further, 14 fungicides were analyzed to check the inhibitory effect on isolated pathogens.

## Materials and methods

### Sample collection and isolation

Samples were collected during plum disease surveys conducted between 2019 and 2020, from affected orchards in Yingmaili Township (39° 31′ 11″ N 76° 55′ 17″ E), Jiashi County, Kashgar Prefecture, Xinjiang, China. We screened out 15 orchards with disease symptoms, and a total of 28,972 fruits were investigated. A total of 10 trees were observed in each orchard, and a 5-point sampling method was used for the observation (Ni et al., 2008). Disease incidence for each orchard was calculated by observing diseased fruits/total number of fruits observed. A total of 200 diseased fruits were collected and brought to the laboratory. Among them, 70 diseased fruits with typical diseased symptoms were separated for fungal isolation. When conidia were visible, single-spore isolation was performed (Zhang et al., 2013; Brahmanage et al., 2020). Alternatively, diseased tissues were grown on potato dextrose agar (PDA) for tissue isolation, as described by Raza et al. (2019). A total of 37 strains of *Mucor* species were isolated, and mycelial plugs were stored in 2 mL tubes for long-term storage at 4°C under sterile water. Dry and living cultures were deposited in the Herbarium of Microbiology, Academia Sinica (HMAS), and China General Microbiological Culture Collection Center (CGMCC), respectively. Taxonomic novelty description and nomenclature were deposited in MycoBank.

### Culture description

Observations of morphological features were made on 4- to 7-day-old fungal colonies incubated at room temperature (28°C) under near-ultraviolet (near-UV) light with 12-h photoperiod and 12-h darkness. A color guide by Kornerup and Wanscher (1967) was used to describe colony color on PDA. The morphological characters were photographed using a Nikon Eclipse Ci-L light microscope (Yokohama, Japan) and an Oplenic D2000 digital camera (USA). Columellae, chlamydospore, sporangia, sporangiophore, and sporangiospores were also observed on slides mounted in 100% lactic acid.

### Genomic DNA extraction, polymerase chain reaction amplification, and sequencing

The genomic deoxyribonucleic acid (gDNA) was extracted using the cetyltrimethylammonium bromide (CTAB) method (Doyle, 1990). Amplification of the internal transcribed spacer (ITS) of isolates was conducted using primer pairs ITS 1 forward (5 TCCGTAGGTGAACCTGCGG-3) and ITS 4 reverse (5 TCCTCCGCTTATTGATATGC-3) (Khan and Javaid, 2022). An initial basic local alignment search tool (BLASTn) analysis was conducted to screen *Mucor* species based on their ITS sequences. *Mucor* strains were also amplified and sequenced for a fragment of 28S rRNA gene with primer pairs NL1 and NL4 (Raza et al., 2019). Each locus was amplified using the polymerase chain reaction (PCR) protocol described by Hurdeal et al. (2021) and Zhao et al. (2023). The PCR reaction was performed in a 25-μL reaction volume using a 15-μL rapid Taq master mix (Vazyme, Nanjing, China), 0.1-mM primers, and 10-ng gDNA. PCR was performed in the following conditions: predenaturation at 95°C for 5 min; denaturation at 95°C for 30 s; annealing at 55°C (for ITS) or 56°C (for large subunit [LSU]) for 40 s; extension at 72°C for 45 s, 35 cycles; and elongation at 72°C for 10 min. The PCR products were detected by 1% agarose gel electrophoresis, and sequencing was done by Sangong Bioengineering (Shanghai) Co., Ltd.

### Phylogenetic analysis

Phylogenetic relationship and taxonomic distinction for novel species were determined using genetic markers recommended in a recent bibliography of the genus *Mucor* (Hurdeal et al., 2021; Zhao et al., 2023). A sequence assembly was performed, and necessary corrections were made manually wherever necessary using BioEdit 7.2.5 (Hall, 1999). Bayesian inference (BI) and maximum likelihood (ML) analyses were employed to reconstruct the phylogeny, respectively, with MrBayes 3.2.7 (Ronquist et al., 2012) and RAxML 8.2.10 (Stamatakis, 2014). Based on the Akaike information criterion, MrModeltest 2.3 (Nylander, 2004) was used to estimate the best-fit evolutionary models for the two-locus dataset. The posterior probability (PP) distribution convergence was ensured by running 6,000,000 generations of Markov chain Monte Carlo (MCMC) with a random seed and a stopval = 0.01 MCMC algorithm of four chains. Based on the 50% majority rule and removing the first 25% of the trees sampled, we calculated consensus trees based on the 50% majority rule and PP. It was considered significant if the PP value was greater than 0.95. Selected bootstrap replicates were 1,000, and bootstrap support (BS) ≥70 was considered significant (Li et al., 2023). Sequences generated in this study were deposited in GenBank, and their accession numbers can be found in Table 1.

**Table 1:** Reference specimens and their GenBank accession numbers were used for phylogenetic analysis in this study.

Strain name	Voucher number	ITS	LSU	References
*Backusella dispersa*	CBS 195.28	JN206271	JN206530	Urquhart et al. (2020)
*Begonia grandis*	CBS 186.87 **T**	NR_103648	JN206527	Walther et al. (2013)
*M. abortisporangium*	CGMCC 3.16133 **T**	OL678180	–	Zhao et al. (2023)
*M. abundans*	CBS 388.35 **NT**	JN206111	NG_063979	Walther et al. (2013); Vu et al. (2019)
*M. aligarensis*	CBS 993.70 **T**	NR_103634	NG_057920	Walther et al. (2013); Schoch et al. (2014)
*M. amethystinus*	CBS 526.68	JN206015	JN206426	Walther et al. (2013)
*M. amphibiorum*	CBS 763.74 **T**	NR_103615	NG_057877	Vitale et al. (2012); Schoch et al. (2014)
*M. amphisporus*	CGMCC 3.16134 **T**	OL678181	–	Zhao et al. (2023)
*M. ardhlaengiktus*	CBS 210.80 **ET**	NR_152960	NG_069778	Walther et al. (2013); Vu et al. (2019)
*M. atramentarius*	CBS 202.28 **T**	MH854979	JN206418	Walther et al. (2013); Vu et al. (2019)
*M. azygosporus*	CBS 292.63 **T**	NR_103639	NG_057928	Walther et al. (2013): Schoch et al. (2014)
*M. bacilliformis*	CBS 251.53 **T**	NR_145285	NG_057916	Walther et al. (2013)
*M. bainieri*	CBS 293.63 **IsoT**	NR_103628	JN206424	Walther et al. (2013); Schoch et al. (2014)
*M. breviphorus*	CGMCC 3.16135 **T**	OL678183	–	Zhao et al. (2023)
*M. brunneolus*	CGMCC 3.16136 **T**	OL678184	–	Zhao et al. (2023)
*M. caatinguensis*	URM 7223 **T**	KT960377	KT960371	Li et al. (2016)
*M. changshaensis*	CGMCC 3.16137 **T**	OL678185	–	Zhao et al. (2023)
*M. chiangraiensis*	MFLUCC 21–0042 **T**	MZ433253	NG_088246	Hurdeal et al. (2021)
*M. chlamydosporus*	CGMCC 3.16138 **T**	OL678187	–	Zhao et al. (2023)
*M. chuxiongensis*	NYNU-174111 **T**	MG255732	NG_228784	Chai et al. (2019)
*M. circinatus*	URM7218	KY008576	KY008571	Lima et al. (2017)
*M. circinelloides*	CBS 195.68	JN205961	NG_055735	Vitale et al. (2012); Walther et al. (2013)
*M. corticola*	CBS 362.68	JN206132	JN206449	Walther et al. (2013)
*M. ctenidius*	CBS 293.66 **IsoT**	MH858796	JN206417	Walther et al. (2013); Vu et al. (2019)
*M. donglingensis*	CGMCC 3.16139 **T**	OL678190	–	Walther et al. (2013)
*M. durus*	CBS 156.51	NR_145295	NG_057918	Walther et al. (2013); Borkar (2021)
*M. endophyticus*	CBS 385.95	NR_111661	NG_057970	Schoch et al. (2014)
*M. exponens*	CBS 141.20	MH854686	JN206441	Walther et al. (2013); Vu et al. (2019)
*M. falcatus*	CBS 251.35	NR_103647	NG_057931	Walther et al. (2013); Schoch et al. (2014)
*M. flavus*	CBS 230.35 **T**	JN206061	JN206464	Walther et al. (2013)
*M. floccosus*	CGMCC 3.16140 **T**	OL678192	–	Zhao et al. (2023)
*M. fusiformisporus*	CGMCC 3.16141 **T**	OL678194	–	Zhao et al. (2023)
*M. genevensis*	CBS 114.08 **T**	NR_103632	NG_057971	Schoch et al. (2014)
*M. gigasporus*	CBS 566.91	NR_103646	NG_057926	Walther et al. (2013); Schoch et al. (2014)
*M. griseocyanus*	CBS 116.08 **T**	NR_126136	NG_056283	Walther et al. (2013)
*M. guiliermondii*	CBS 174.27	NR_103636	NG_057923	Walther et al. (2013); Schoch et al. (2014)
*M. heilongjiangensis*	CGMCC 3.16142 **T**	OL678198	–	Zhao et al. (2023)
*M. heterogamus*	CBS 338.74	JN206169	JN206488	Walther et al. (2013)
*M. hiemalis*	CBS 201.65	JX976246	NG_057968	Lu et al. (2013)
*M. hemisphaericum*	CGMCC 3.16143 **T**	OL678200	–	Zhao et al. (2023)
*M. homothallicus*	CGMCC 3.16144 **T**	OL678201	–	Zhao et al. (2023)
*M. hyalinosporus*	CGMCC 3.16145 **T**	OL678203	–	Zhao et al. (2023)
*M. indicus*	CBS 226.29	NR_077173	NG_057878	Vitale et al. (2012); Schoch et al. (2014)
*M. irregularis*	CBS 103.93 **T**	JN206150	NG_056285	Lu et al. (2013); Hurdeal et al. (2021)
*M. japonicus*	CBS 154.69 **NT**	JN206158	JN206446	Walther et al. (2013)
*M. koreanus*	CNUFC-EML-QT1	KT936259	NG_068529	Li et al. (2016)
*M. laxorrhizus*	CBS 143.85	NR_103642	NG_057914	Walther et al. (2013); Schoch et al. (2014)
*M. lobatus*	CGMCC 3.16146 **T**	OL678204	–	Zhao et al. (2023)
*M. lusitanicus*	CBS 108.17 **ET**	JN205980	NG_056279	Alvarez et al. (2011); Walther et al. (2013)
*M. luteus*	CBS 243.35	JX976254	NG_057969	Lu et al. (2013)
*M. megalocarpus*	CBS 215.27	NR_145286	NG_057925	Walther et al. (2013)
*M. merdicola*	URM 7222 **T**	KT960374	KT960372	Li et al. (2016)
*M. merdophylus*	URM 7908 **T**	MK775467	MK775466	Lima et al. (2020)
*M. minutus*	CBS 586.67 **T**	NR_152958	JN206463	Walther et al. (2013)
*M. moelleri*	CBS 444.65 **T**	MH858663	MH870304	Vu et al. (2019)
*M. moniliformis*	CGMCC 3.16147 **T**	OL678206	–	Zhao et al. (2023)
*M. mousanensis*	CBS 999.70	NR_103629	NG_057912	Walther et al. (2013); Schoch et al. (2014)
*M. mucedo*	CBS 640.67	JN206085	MH870785	Walther et al. (2013); Vu et al. (2019)
*M. multiplex*	CBS 110662	NR_111662	NG_057924	Walther et al. (2013); Schoch et al. (2014)
*M. nederlandicus*	CBS 735.70	JN206176	JN206503	Walther et al. (2013)
*M. nidicola*	H13	KX375786	KX375769	Hurdeal et al. (2021)
*M. odoratus*	CBS 130.41	NR_145287	NG_057927	Walther et al. (2013)
*M. orantomantidis*	CNUFC-MID1–1 **T**	MH594737	MH591457	Phookamsak et al. (2019)
*M. orientalis*	CGMCC 3.16148 **T**	OL678208	–	Zhao et al. (2023)
*M. parviseptatus*	CBS 417.77	JN206108	JN206453	Walther et al. (2013)
*M. pernambucoensis*	URM 7640 **T**	MH155323	MH155322	Li et al. (2016)
*M. piriformis*	CBS 169.25	NR_103630	NG_057874	Vitale et al. (2012); Schoch et al. (2014)
*M. plasmaticus*	CBS 177.46	JN206076	MH867680	Walther et al. (2013); Vu et al. (2019)
*M. plumbeus*	CBS 666.66	MH858910	MH870586	Vu et al. (2019)
*M. prayagensis*	CBS 816.70	JN206188	MH871756	Walther et al. (2013); Vu et al. (2019)
*M. pseudocircinelloides*	CBS 541.78 **T**	JN206013	JN206431	Wagner et al. (2019)
	XY07713	OL620144	–	Zhao et al. (2023)
*M. pseudolusitanicus*	CBS 540.78 **T**	MF495059	NG_073591	Wagner et al. (2019)
	CBS 543.80	MF495060	–	Wagner et al. (2019)
	2203.2	OR885026	–	–
	4–4	OR879995	–	–
*M. racemosus*	CBS 260.68	JN205898	MH870843	Walther et al. (2013)
*M. radiatus*	CGMCC 3.16149 **T**	OL678209	–	Zhao et al. (2023)
*M. ramosissimus*	CBS 135.65 **NT**	NR_103627	NG_056280	Alvarez et al. (2011); Schoch et al. (2014)
*M. rhizosporus*	CGMCC 3.16150 **T**	OL678211	–	Zhao et al. (2023)
*M. robustus*	CGMCC 3.16151 **T**	OL678212	–	Zhao et al. (2023)
*M. rudolphii*	WU 35867	KT736104	–	Voglmayr and Clémençon (2016)
*M. saturninus*	CBS 974.68 **T**	NR_103635	JN206458	Walther et al. (2013); Schoch et al. (2014)
*M. septatum*	URM 7364 **T**	KY849814	KY849816	De Souza et al. (2018)
*M. silvaticus*	CBS 509.66	JN206123	MH870514	Walther et al. (2013); Vu et al. (2019)
*M. sino-saturninus*	CGMCC 3.16152 **T**	OL678215	–	Zhao et al. (2023)
*M. aseptatophorus*	MFLU 21–0040 **T**	MZ433252	MZ433249	Hurdeal et al. (2021)
*M. souzae*	URM 7553 **T**	KY992878	NG_067797	Crous et al. (2018)
*M. stercorarius*	CNUFC-UK2–1 **T**	KX839689	KX839685	Tibpromma et al. (2017)
*M. strictus*	CBS 576.66	JN206037	NG_076700	Walther et al. (2013)
*M. thermorhizoides*	CBS 149760 **T**	OQ034234	–	Abramczyk et al. (2024)
*M. ucrainicus*	CBS 674.88	JN206192	JN206507	Walther et al. (2013)
*M. variicolumellatus*	CBS 236.35 **T**	JN205979	JN206422	Walther et al. (2013)
*M. variisporus*	CBS 837.70	NR_152951	NG_057972	Hurdeal et al. (2021)
***M. xinjiangensis***	**19Z3 = CGMCC 3.27539 = 19Z3 T**	**PP905027**	**PP905032**	**Present study**
	**39Z29**	**PP905028**	**PP905033**	**Present study**
	**33**	**PP905029**	–	**Present study**
	**16Z2**	**PP905030**	–	**Present study**
	**21Z3**	**PP905031**	–	**Present study**
*M. yunnanensis*	ZHKUCC 22–0110 **T**	ON921544	ON921546	Gajanayake et al. (2023)
*M. zonatus*	CBS 148.69	NR_103638	NG_057917	Walther et al. (2013); Schoch et al. (2014)
*M. zychae*	CBS 416.67	NR_103641	NG_057930	Walther et al. (2013); Schoch et al. (2014)

**T**, **ET**, **NT**, and **IsoT** denote type, ex-epitype, ex-neotype, and ex-isotype cultures, respectively. ITS, internal transcribed spacer; LSU, large subunit. Novel taxon is indicated in bold.

### Pathogenicity test

Two representative strains of *Mucor* species (CGMCC 3.27539 and 39Z29) were tested for pathogenicity (Hussain et al., 2016). Selected fresh and healthy new plum fruits were surface sterilized with 75% ethanol, and then a 4-mm diameter wound was made in the middle of the fruit using a wound retractor. Mycelial plugs were taken from the margin of the growing colonies of isolates using a 4-mm diameter cork borer. Fresh wounds were inoculated by placing mycelial plugs into the wounds. A plug of PDA with no fungal growth was included as a control. The inoculated fruits were arranged in a sterile humidity (65–70%) chamber for 7 days, and fruits were observed every day. Twenty fruits were inoculated with two representative strains and repeated 3 times. After the incubation period, disease lesions were measured, and pathogens were re-isolated (Song et al., 2020). The morphological characteristics and ITS sequences of the re-isolated fungus were compared to those of the original strains. The inoculation experiment was conducted 2-times to ensure reliability.

### Fungicides and adjuvants against pathogen

A total of 14 fungicides, including 10 fungicides and 4 adjuvants (Table 2), were tested *in vitro* against the pathogen (type strain) (Hussain et al., 2014). The fungicides selected in this study were registered under the Pesticide Inspection Institute, Ministry of Agriculture and Rural Affairs, China.^1^ The fungicide solutions were prepared according to the label instructions provided by their manufacturers (Table 2). The recommended concentration was diluted, and 1 mL was added to 49 mL PDA medium and poured into a 9 cm Petri dish. An equal volume of sterile water was added to the control Petri dish. A plug of mycelium (5 mm) was inoculated into a petri dish containing fungicides and kept at 28°C for 7 days with daily checks (Wagner et al., 2019). Each treatment was replicated 4 times. The colony diameter was measured, and the inhibition rate was calculated as follows:


$$\begin{array}{l} \mathrm{Mycelial growth inhibition}\;\left(\%\right)\\ =\left[\begin{array}{l} \mathrm{average}\;\;\mathrm{growth diameter}\;\;\mathrm{of control colonies}\\ -\mathrm{average}\;\;\mathrm{growth diameter}\;\;\mathrm{of treated colonies}/\\ \mathrm{growth diameter of control colonies} \end{array}\right]\times 100 \end{array}$$

**Table 2:** Fungicides and fungicides/adjuvants mixture tested against *Mucor xinjiangensis* in this study.

Commercial name (Chinese pinyin)	Active ingredient (AC)	AC rate (g/L)	Dosage form	Recommended dosage by company	Production company
Lóngdēng tǒng wàng	Carbendazim	500	Suspension concentrate	120–150 mL/acre	Jiangsu Longdeng Chemical Co., Ltd.
Bǎi jūn qīng	Chlorothalonil	750	Wettable powder	150–200 g/acre	Shaanxi Hengtian Biological Agriculture Co., Ltd.
Shì jié	Difenoconazole	200	Emulsion in water	30–40 mL/acre	Shaanxi Thompson Biotechnology Co. Ltd.
Yǐ mì fēn	Ethirimol	250	Suspension concentrate	65–95 mL/acre	Jiangxi Heyi Chemical Co., Ltd.
Yībiàn jìng	Ethylicin	800	Emulsifiable concentrate	25–30 g/acre	Henan Kebang Chemical Co., Ltd.
Fú guī zuò	Flusilazole	400	Emulsifiable concentrate	10–20 mL/acre	Beijing Agonon Biopharmaceutical Co. Ltd.
Ruì pǔ shēng	Mancozeb	800	Wettable powder	150–240 g/acre	Shandong Baishiwei Crop Protection Co., Ltd.
Bǎo fēng	Pyraclostrobin	250	Suspending agent	60–120 mL/acre	Anyang City Ruipu Agrochemical Co., Ltd.
Wù zuò chún	Tebuconazole	430	Suspension concentrate	20–30 mL/acre	Xi ‘an Dingsheng Bio-Chemical Co., Ltd.
Dà fēng tuō	Thiophanatemethyl	500	Suspension concentrate	100–150 mL/acre	Shaanxi Hengtian Biological Agriculture Co., Ltd.
Měi shí lè	Difenoconazole + azoxystrobin	150; 250	Suspension concentrate	30–40 mL/acre	Qingdao Hansheng Biotechnology Co., Ltd.
Xīng líng	Prochloraz + aprodione	100; 100	Suspension concentrate	200–240 mL/acre	Shaanxi Hengrun Chemical Industry Co., Ltd.
Yōu zé shí	Pyraclostrobin + aebuconazole	250; 430	Microemulsion	60–120 mL/acre	Qingdao Hansheng Biotechnology Co., Ltd.
Shè xǐ	Trifloxystrobin + aebuconazole	250; 500	Water dispersible granule	12–15 g/acre	Qingdao Odis Biotechnology Co., Ltd.

In addition, the average growth rate of mycelium in each treatment was used to screen out fungicides that were effective, and the half maximal effective concentration (EC_50_) value was calculated to measure the toxicity of the agent, using the recommended concentration as the center (used as control). A total of five concentrations were set with four replicates for each concentration.

### Statistical analysis

Each measurement was repeated at least 3 times. Dunnett tests were used to compare mean values based on univariate analysis of variance (ANOVA) with Statistical Package for the Social Sciences (SPSS) version 19.0 (IBM) software. Different letters above the bars indicate statistical differences (*p* < 0.05).

## Results

### Disease symptoms and incidence

**Figure 1:**
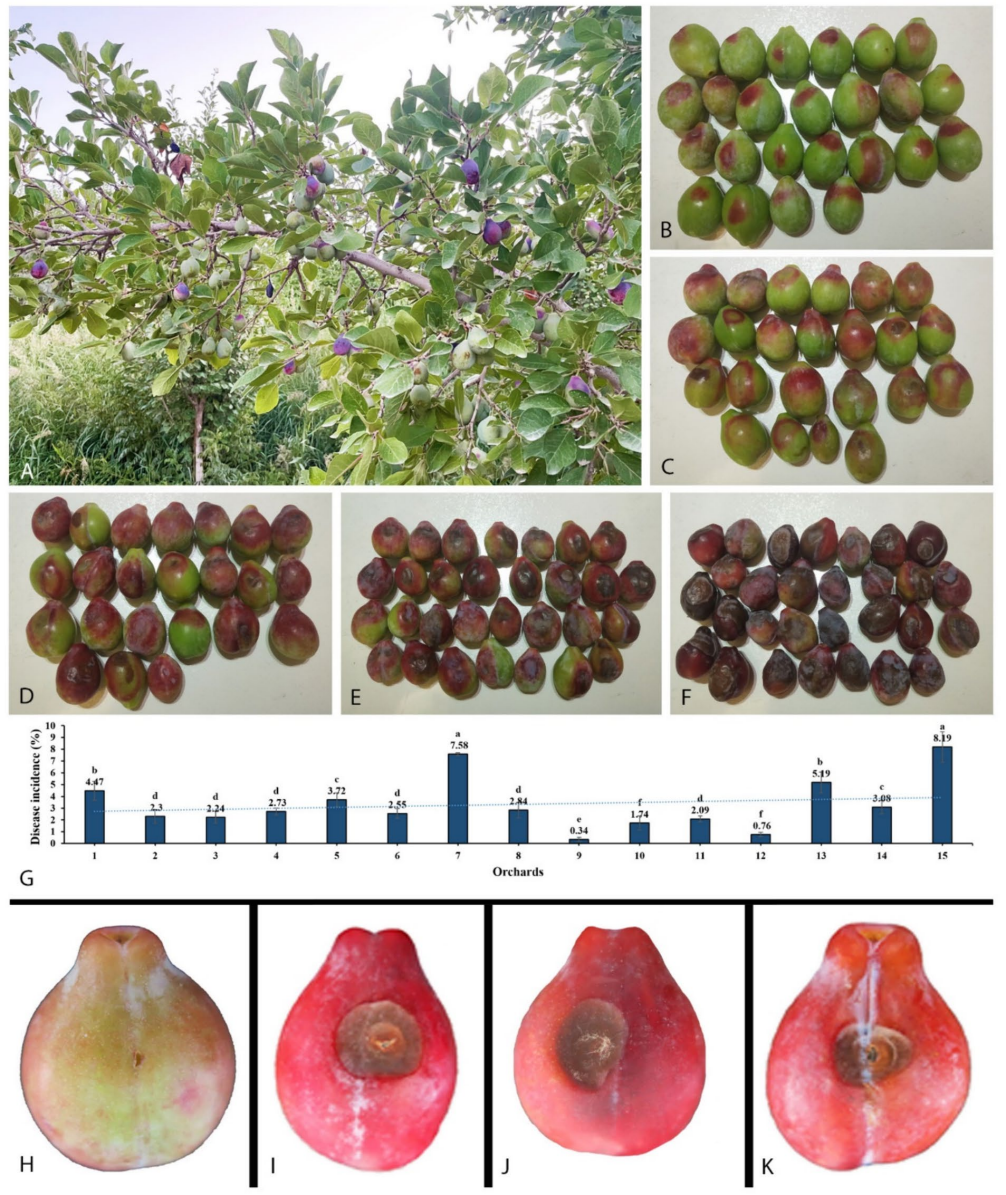
Disease development in the field on plum tree and pathogenicity test. **(A)** Infected plant. **(B−F)** Gradually disease expansion on fruit collected from different orchards. **(G)** Disease incidence in 15 orchards. **(H)** Control. **(I)** Inoculated with *Mucor xinjiangensis*, strain CGMCC 3.27539. **(J)** Inoculated with *M. xinjiangensis*, strain 39Z29. **(K)** Inoculated directly with diseased plum tissue collected from the field.

The initial symptom of the newly observed disease was small, scattered red spots appearing after the onset of the fruit (Figures 1A–F). It was found that the disease spreads swiftly when the temperature rises, resulting in brown spots that sag and expand around, wrinkle, and harden, and eventually lead to fruit falling from the tree. At high humidity, especially early in the morning, white mycelia were observed on infected fruits. In our investigation, we found that a total of 1,032 fruits were diseased out of 28,972. The disease incidence caused by *Mucor* species for each orchard was between 0.31 and 7.63% (Figure 1G).

### Koch’s postulates for pathogenicity test

A total of 70 diseased fruit samples with typical disease symptoms were collected, from which fungi were isolated in 21 samples. This yielded 60 fungal isolates, of which 37 were morphologically identified as *Mucor* species, representing 61.67% of the total isolates. Plum fruits inoculated with two representative strains showed brown rot symptoms of 1.5–2 cm after 7 days. The symptoms first consisted of brown spots that expanded. As brown spots develop, the infected area becomes wrinkled and hard, then fades to dark brown. Infected fruits were found to have white mycelia. Fruits infected with the representative strains developed similar symptoms to those observed in the field. There were no visible symptoms of brown rot on the non-inoculated control fruit (Figures 1H–K). Strains were recovered from the inoculated diseased fruit symptoms, which were conspecific to the original isolates from the natural diseased fruits, based on microscopic characteristics and 100% similarity in ITS and LSU sequences.

### Phylogenetic analyses

**Figure 2:**
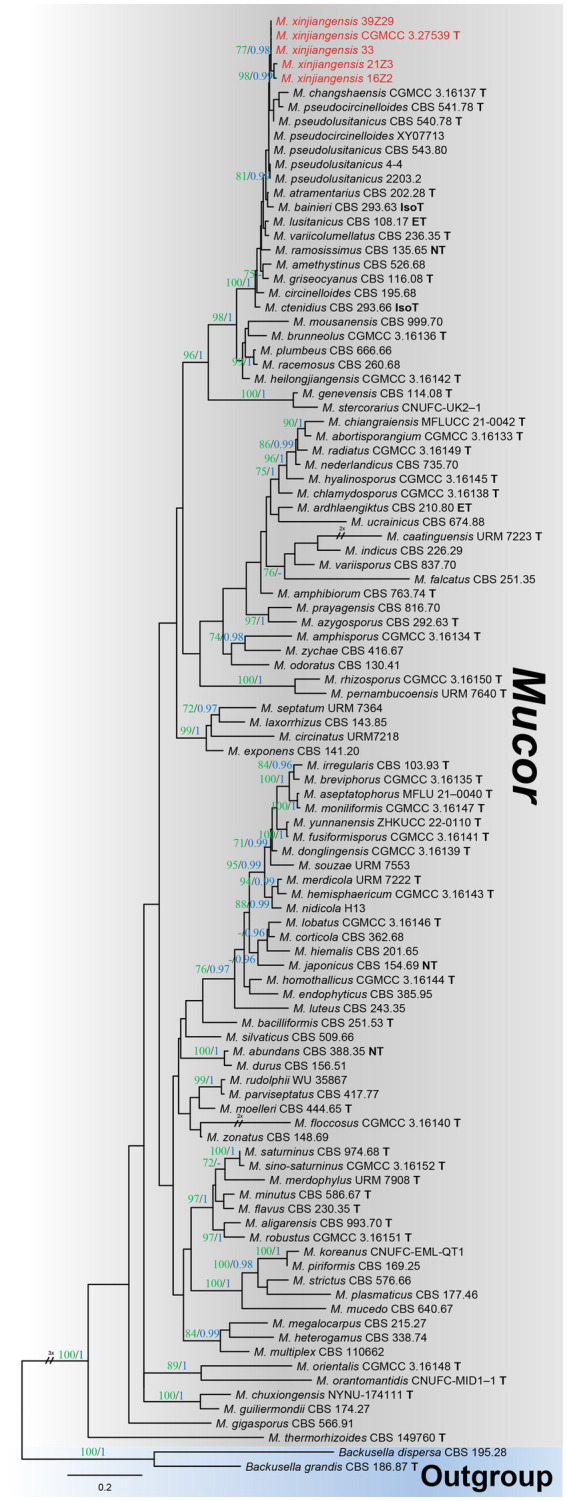
Maximum likelihood (ML) phylogenetic tree inferred from a two-locus concatenated alignment (ITS and LSU). Bootstrap values >70% for ML in green and posterior probability (PP) >0.95 in blue were added on the above and below the branch length (ML/PP). The type, epitype, neotype, and isotype strains were indicated in bold with T, ET, NT, and IsoT, respectively. The strains introduced in this study are represented in red. The tree is rooted using *Backusella dispersa* (CBS 195.28) and *Begonia grandis* (CBS 186.87).

Among the total *Mucor* isolates, five strains were randomly selected basis of different collection sites (fields) and successfully amplified with single ITS fragments. A preliminary comparison of ITS sequences via BLASTn search showed that each strain belongs to *Mucor* but representative strains (CGMCC 3.27539 and 39Z29) were most closely related to *Mucor pseudolusitanicus* (MF495059; 96% query cover and 98% identity and MF495059; 98% query cover and 99% identity, respectively). By analyzing the combined dataset of ITS and LSU loci, strains collected from diseased plums were further identified. A total of 93 reference sequences were used, including 91 *Mucor* species, and two species of the *Backusella* were used as outgroups, retrieved from GenBank (Table 1). In the concatenated alignment of five *Mucor* strains and 93 reference taxa, 957 distinct alignment patterns and 29.22% proportion of gaps and completely undetermined characters. Based on the combined ITS and LSU phylogenetic analyses, our strains formed an exclusive and well-supported clade (93/0.98 for ML/PP) (Figure 2). There was a strong correlation between the topologies of the individual gene trees and the concatenated tree, indicating that both strains recovered from brown spots on plum fruit were distinct species of *Mucor*.

### Taxonomy

**Figure 3:**
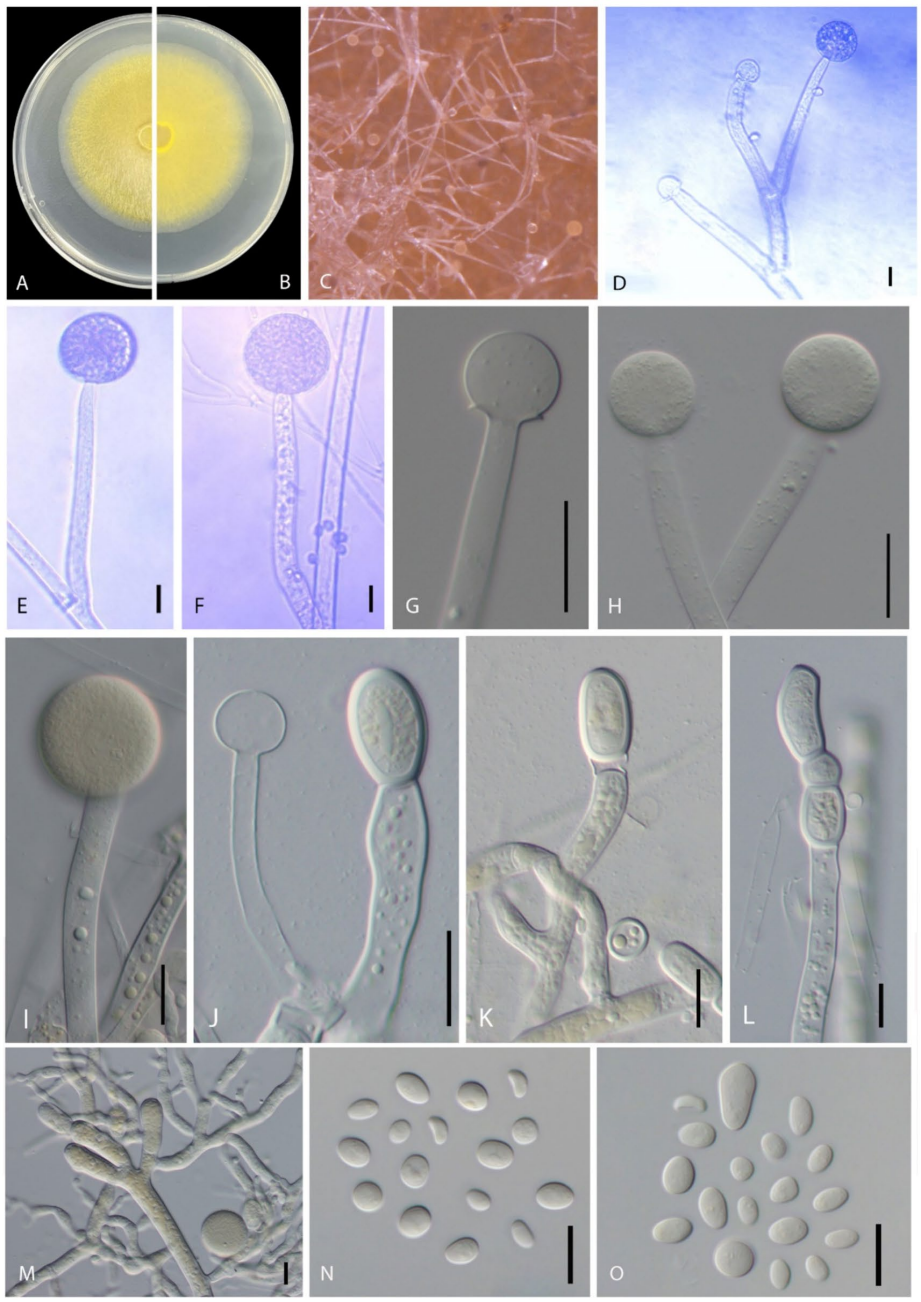
Disease symptoms and morphological characteristics of *Mucor xinjiangensis* (CGMCC 3.27539). **(A,B)** Colony on PDA—**(A)** from above and **(B)** from below. **(C)** Sporulation on PDA. **(D,E)** Columellae. **(F–I)** Sporangia. **(J)** Columella and chlamydospores. **(K–M)** Chlamydospores. **(N–O)** Sporangiospores. Scale bars: **(D–F,N)** = 10 μm; **(G–M)** = 20 μm.

***Mucor xinjiangensis*** B. Song & M. Raza, sp. nov. (Figures 3A–O).

MycoBank: MB853833.

*Etymology*: refers to Xinjiang Uyghur Autonomous Region in China from which the holotype was isolated.

*Typification*: China, Xinjiang Uyghur Autonomous Region, Kashgar prefecture, Jiashi county, on *P. domestica* (European plum), July 2019, *B. Song* (HMAS 352969, ex-type living culture 19Z3 = CGMCC 3.27539).

*Morphology*: *Hyphae* smooth, branched, aseptate, hyaline to yellowish, 5.5–13 μm diameter. *Sporangiophore* erects directly from aerial hyphae, small and tall, colorless, simple or 1–2 times sympodially branched, 35–160 μm in length (average = 93.04 ± 40.82 μm), 6–12 μm in diameter (average = 8.70 ± 1.67 μm), branches often subterminal and longer than the main stems, all terminating with a sporangium, non-apophysate below the sporangium. *Sporangia* non-apophysate, globose to slightly depressed globose, 16–42.5 μm in width (average = 26.88 ± 9.73 μm), the wall is slowly dissolving or broken, grayish brown. *Columellae* globose or subglobose, 11–19.5 μm width (average = 16.35 ± 2.42 μm), hyaline or pale orange–brown, no collar. *Sporangiospores* variable in shape, ellipsoidal to obovoid, 4–11 × 3–7 μm (average = 6.89 ± 1.13 × 4.83 ± 0.63 μm) wide, colorless. *Chlamydospore* occurring in vegetative hyphae, smooth, thin walled, intercalary, single, in pairs or chains, globose, subglobose, 15–35 × 12–19 μm width (average = 21.45 ± 6.09 × 15.37 ± 1.93 μm). *Rhizoids* present. *Zygospores* not observed.

*Other specimens examined*: China, Xinjiang Uyghur Autonomous Region, Kashgar prefecture, Jiashi county, on *P. domestica* (European plum), July 2019, *B. Song*, living culture 39Z29.

*Cultural characteristics*: *Colonies* on PDA are fast growing, reaching 6.8 cm in diameter in 2 days after incubation at 28 ± 1°C, colony medium, slightly raised with an erose edge, rough surface, effuse, well-defined margin; colony from above; dull, medium, whitish to pale yellow, later blackish; from below, pale yellow; not producing pigment in PDA media. Sporulate on PDA.

*Notes*: Five strains of *Mucor xinjiangensis* clustered together and closely related to *Mucor changshaensis*, *Mucor pseudocircinelloides*, and *Mucor pseudolusitanicus*, but type isolate (CGMCC 3.27539) differs in producing smaller sized sporangia (23.5–52 μm in *M. changshaensis*, up to 90-μm in diameter in *M. pseudocircinelloides*, up to 75-μm in diameter in *M. pseudolusitanicus*, and up to 16–42.5 μm in *M. xinjiangensis*), columellae (10–28.5 × 10.5–28 μm in *M. changshaensis*, 27–46 × 34–58 μm in *M. pseudocircinelloides*, 35–52 μm in *M. pseudolusitanicus*, and 11–19.5 μm in *M. xinjiangensis*), and larger chlamydospores (8.5–20 × 7–16.5 μm in *M. changshaensis*, 2.3–26.7 × 9.8–17.4 μm in *M. pseudocircinelloides*, 10.4–19.7 × 6.7–15.4 μm in *M. pseudolusitanicus*, and 15–35 × 12–19 μm in *M. xinjiangensis*). *M. pseudocircinelloides* and *M. pseudolusitanicus* produce hyaline to pale brown sporangia, while *M. xinjiangensis* produces hyaline to grayish brown sporangia (Wagner et al., 2019). In the case of *M. changshaensis*, these sporangia are light brown to black (Zhao et al., 2023). Our collection (CGMCC 3.27539) ITS loci are 3.2% (18 out of 560 bp) different from both *M. changshaensis* and *M. pseudocircinelloides*, while 0.8% (5 out of 560 bp) different from those of *M. pseudolusitanicus*. For LSU, this difference is 1.32% (9 out of 679 bp), with both *M. pseudocircinelloides* and *M. pseudolusitanicus* and LSU of *M. changshaensis* is not available. Furthermore, *M. xinjiangensis* produces a white to pale yellow color on PDA compared to those *M. changshaensis* (light to strontian yellow), *M. pseudocircinelloides* (white to pale brown, reverse uncolored), and *M. pseudolusitanicus* (white to light gray, reverse uncolored).

### Evaluation of fungicides and fungicide adjuvants

**Figure 4:**
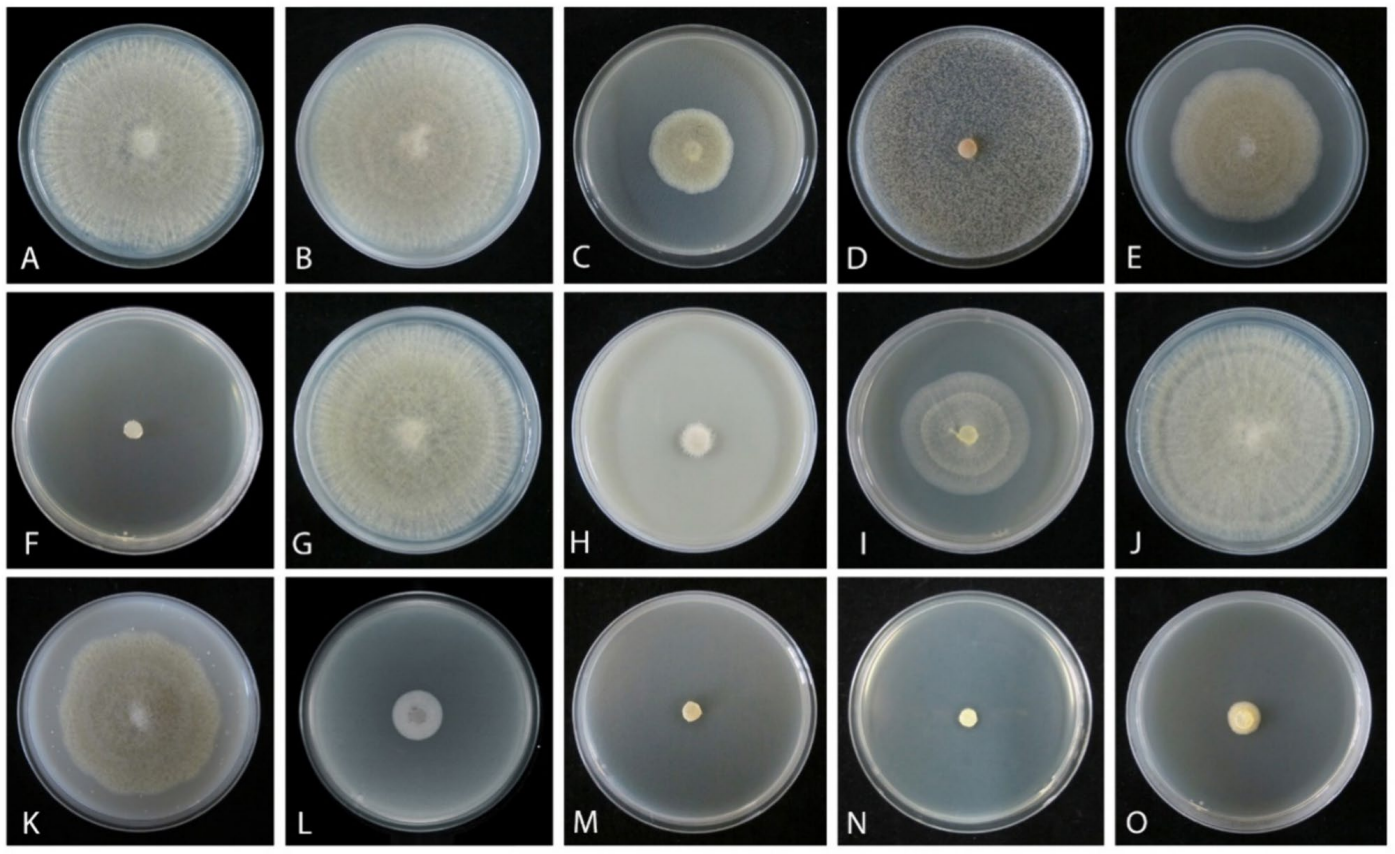
Mycelial growth inhibition of *Mucor xinjiangensis* (CGMCC 3.27539) from different fungicides and fungicide adjuvants with recommended dosage, after 5 days. **(A)** Control. **(B)** Carbendazim (500 g/L). **(C)** Chlorothalonil (750 g/L). **(D)** Difenoconazole (PDA texture differentiation was caused by fungicide addition) (200 g/L). **(E)** Ethirimol (250 g/L). **(F)** Ethylicin (800 g/L). **(G)** Flusilazole (400 g/L). **(H)** Mancozeb (800 g/L). **(I)** Pyraclostrobin (250 g/L). **(J)** Tebuconazole (430 g/L). **(K)** Thiophanatemethyl (500 g/L). **(L)** Difenoconazole + Azoxystrobin (150, 250 g/L). **(M)** Prochloraz + Iprodione (100, 100 g/L). **(N)** Pyraclostrobin + tebuconazole (250, 430 g/L). **(O)** Trifloxystrobin + tebuconazole (250, 500 g/L).

**Figure 5:**
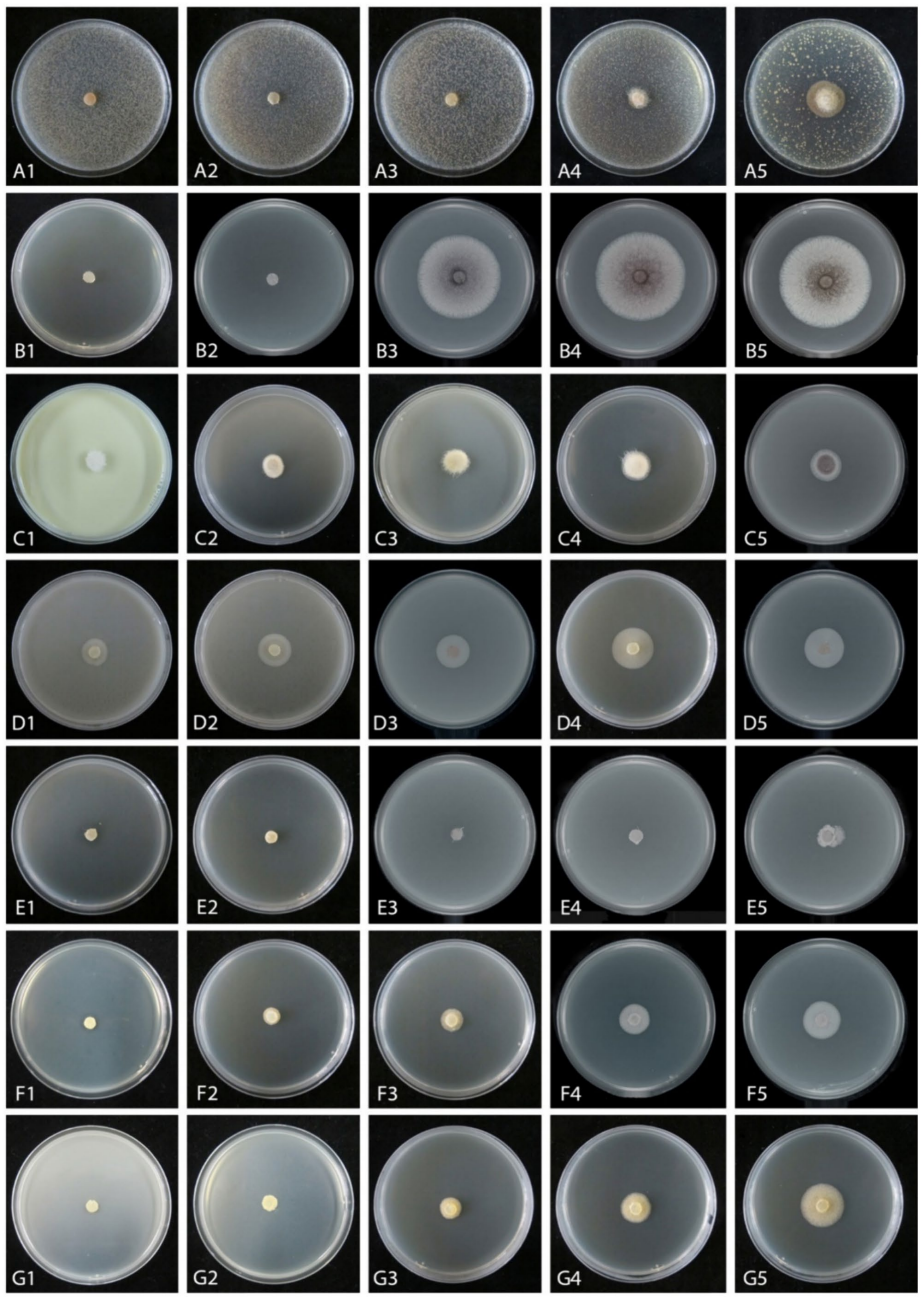
Mycelial growth inhibition of *Mucor xinjiangensis* (CGMCC 3.27539) from different fungicides and fungicide adjuvants at different dilution concentrations after 5 days. **(A1–A5)** Difenoconazole diluted concentration (PDA texture differentiation was caused by fungicide addition) (A1. at 3,000; A2. at 5,000; A3. at 7,000; A4 at 10,000; A5. at 20,000). **(B1–B5)** Ethylicin diluted concentration (B1. at 2,000; B2. at 3,000; B3. at 4,000; B4 at 5,000; B5. at 6,000). **(C1–C5)** Mancozeb (C1. at 900; C2. at 1,500; C3. at 3,000; C4 at 4,000; C5. at 6,000). **(D1–D5)** Difenoconazole + azoxystrobin diluted concentration (D1. at 300; D2. at 600; D3. at 1,500; D4. at 2,400; D5. at 3,000). **(E1–E5)** Prochloraz + iprodione diluted concentration (E1. at 500; E2. at 1,000; E3. at 2,000; E4. at 4,000; E5. at 8,000). **(F1–F5)** Pyraclostrobin + tebuconazole diluted concentration (F1. at 2,000; F2. at 6,000; F3. at 8,000; F4 at 10,000; F5. at 15,000). **(G1–G5)** Trifloxystrobin + tebuconazole diluted concentration (G1. at 1,000; G2. at 2,000; G3. at 4,000; G4. at 8,000; G5. at 10,000).

**Figure 6:**
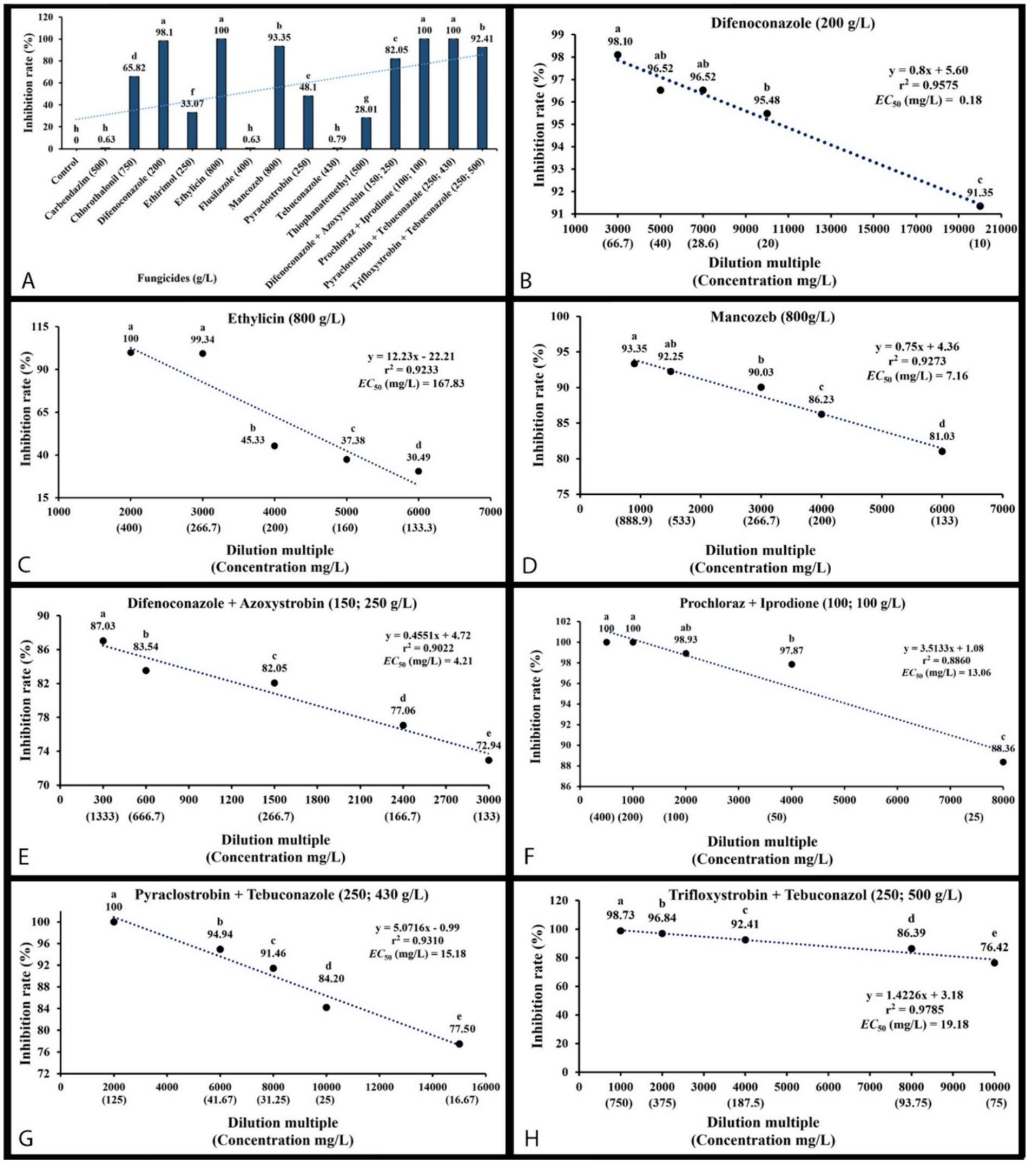
Mycelial growth inhibition percentage of *Mucor xinjiangensis* (CGMCC 3.27539) to fungicides and fungicide adjuvants and correlation of the sensitivity (EC_50_ value) to them. **(A)** Inhibition of *M. xinjiangensis* to a total of 14 fungicides and fungicide adjuvants. **(B)** EC_50_ value to difenoconazole. **(C)** EC_50_ value to ethylicin. **(D)** EC_50_ value to mancozeb. **(E)** EC_50_ value to difenoconazole + azoxystrobin. **(F)** EC_50_ value to prochloraz + iprodione. **(G)** EC_50_ value to pyraclostrobin + tebuconazole. **(H)** EC_50_ value to trifloxystrobin + tebuconazole.

An *in vitro* sensitivity test (Bras and Deloron, 1983) of total 14 fungicides, including 10 fungicides named carbendazim, chlorothalonil, difenoconazole, ethirimol, ethylicin, flusilazole, mancozeb, pyraclostrobin, tebuconazole, and thiophanatemethyl, and 4 fungicide adjuvants including difenoconazole + azoxystrobin, prochloraz + iprodione, pyraclostrobin + tebuconazole, and trifloxystrobin + tebuconazole showed the different results. Our isolated strain (CGMCC 3.27539) was more sensitive to all fungicide adjuvants, and three fungicides, such as difenoconazole, ethylicin, and mancozeb, compared to other fungicides (Figures 4–6). First, ethylicin, prochloraz + iprodione, pyraclostrobin + tebuconazole showed the best inhibitory effect on the growth of the pathogen, and this inhibitory rate was as high as 100% at the recommended concentration. Difenoconazole, mancozeb, and trifloxystrobin + tebuconazole showed growth inhibitory effects of 98.10, 93.35, and 92.41%, respectively. Difenoconazole + azoxystrobin inhibitory effect was more than 80% (82.05%), and chlorothalonil showed general inhibition of 65.82%. Ethirimol, pyraclostrobin, and thiophanatemethyl have small inhibitory effects with inhibition rates of 33.07, 48.1, and 28.01%, respectively (Figures 4A–O). Carbendazim, flusilazole, and tebuconazole have very little effect on the growth of the isolated pathogen.

The effective fungicides and fungicide adjuvants were further tested with different concentrations, including the recommended dosage for virulence determination with the EC_50_ value (Figure 5). There are some differences in the effective medium concentration of EC_50_ value for the seven agents against the *M. xinjiangensis* (Figures 6A–H). Among them, difenoconazole showed the best antifungal effects, the highest toxicity, and the lowest EC_50_ (0.18 mg/L). In the next two, difenoconazole + azoxystrobin and mancozeb were found to have EC_50_ values of 4.21 and 7.16 mg/L, respectively. The antifungal effect of ethylicin was relatively less, with the highest EC_50_ value of 167.83 mg/L. The EC_50_ value of other fungicide adjuvants ranged from about 13–20 mg/L including prochloraz + iprodione (13.06 mg/L), pyraclostrobin + tebuconazole (15.18 mg/L), and trifloxystrobin + tebuconazole (19.18 mg/L).

## Discussion

*Prunus domestica* is widely grown in high temperature and cool humid regions of northwest China (Cao et al., 2014). An unknown brown rot disease on plum fruit was found in several locations in Xinjiang, and its causal agent was unidentified. In the present study, we identified and described the pathogen as a new species, *M. xinjiangensis*. In the combined phylogenetic analysis of ITS and LSU, *M. xinjiangensis* formed a sister clade to *M. pseudocircinelloides* and *M. pseudolusitanicus*. Although *M. xinjiangensis* shares 99% ITS identity with *M. pseudocircinelloides* (XY07713) and *M. pseudolusitanicus* (CBS 543.8), LSU shares 100% ITS identity with *M. pseudolusitanicus* (CBS 540.78), but no LSU blast matches any *M. pseudocircinelloides* strain. By removing the ambiguous sequences of *M. pseudolusitanicus* in combined phylogenetic analysis, we found that *M. pseudolusitanicus* has a wide phylogenetic distribution even within its clade but is different in comparison with its type species (see above notes section). Furthermore, *M. xinjiangensis* is also genetically close to *M. circinelloides* and *M. ctenidius*, which are also pathogenic to plants (Nishijima et al., 2011; Sha and Meng, 2016). Nevertheless, these two species were differentiated from our collection based on morphological characteristics of sporangiophores, sporangia, columellae, and sporangiospores. *M. xinjiangensis* produces relatively larger sporangiophores (35–160 μm in *M. xinjiangensis*, 12–20 μm in *M. circinelloides*, 3–10 μm in *M. ctenidius*), sporangiospores (4–11 × 3–7 μm *M. xinjiangensis*, 4–7 × 3–6.2 μm in *M. circinelloides*, 4–8 × 3.2–6.4 μm in *M. ctenidius*), and smaller sporangia (40–53.5 × 39–53 μm in *M. circinelloides*, 50–70 μm in *M. ctenidius*, 16–42.5 μm in *M. xinjiangensis*), columellae (16–44 × 15–35 μm in *M. circinelloides*, 45–60 × 35–45 μm in *M. ctenidius*, 11–19.5 μm in *M. xinjiangensis*) compared to those of *M. circinelloides* and *M. ctenidius* (Walther et al., 2013).

Among the plum and prunes diseases, the most important postharvest diseases are brown rot, blue mold rot, gray rot, Mucor rot, Rhizopus rot, and bitter rot caused by *Monilinia* species (*Monilinia laxa* or *Monilinia fructicola*), *Penicilliun expansum*, *Botrytis cinerea*, *M. piriformis*, *Rhizopus* spp., *Colletotrichum* spp. (*Colletotrichum gloeosporioides* or *Colletotrichum acutatum*), respectively (Børve and Vangdal, 2007). Usually, these diseases begin with punctured wounds or insect bites. It is important to note, however, that Mucor rot and Rhizopus rot typically share the same symptoms. Due to Mucor rot or Rhizopus rot, the plums become soft, watery, and covered with black spore masses as the infection develops rapidly (Shahnaz et al., 2021; Seethapathy et al., 2022). In Mucor rot, it is often found that fungal structures are stiffer than those of Rhizopus rot, orientated at specific angles to the fruit surface at the time of maturation (Michalltdes, 1990). Rhizopus fruit rot is usually of less importance than the Mucor brown rot in the field, but both can cause important postharvest losses (Dennis and Mountford, 1975). However, the new brown rot disease reported here is the most severe in the field during June and July under high temperatures following continuous rainfall, when plum fruit faces continuous sunshine. The effects of sunburn are slow tree decline and browning of the skin on fruit exposed to too much heat (Racsko and Schrader, 2012; Lal and Sahu, 2017). It might be possible that continuous sunshine toward the premature plum promotes the initial infection (red spots), and then airborne *Mucor* pathogen attacks it and enhances the process of the premature fall of the fruit. Additionally, *M. xinjiangensis* was isolated from symptomatic plum fruit tissues, which showed different symptoms from Rhizopus rot and abiotic stress (sunburn). Due to these reasons, we propose a new name for the disease, “Mucor brown rot,” in order to distinguish it from two well-known biotic and abiotic diseases. Furthermore, healthy plum fruits inoculated with diseased fruit tissue collected from the field and with an isolated strain (*M. xinjiangensis*) also showed the same symptoms.

To date, *M. xinjiangensis* has only been isolated from *P. domestica* among stone fruits. The inoculation and re-isolation tests confirmed that *M. xinjiangensis* is pathogenic against *P. domestica*, and may be even more pathogenic. A range of hosts for *M. xinjiangensis* is unknown at the moment. Other stone fruits should be investigated for *M. xinjiangensis* infection.

It was found that different fungicides and fungicide adjuvants have different inhibition effects on pathogenic fungi (Nita et al., 2007). In the present study, 14 fungicides and their combinations were tested and compared with recommended dosage. These fungicides were commonly available in the Xinjiang market to control plant diseases. Among them, the antifungal rate of seven agents was more than 80%. As broad-spectrum low-toxicity, these fungicides are widely used to control fungal diseases on fruit trees, including apple, grape, peach, pear, and plum, in addition to combination formations. They are mainly classified into triazole (Toda et al., 2021), strobilurin (Balba, 2007), imidazole, benzimidazole, dicarboximidie, protection (chlorthalonil, mancozeb), and the plant bionic pesticide ethylicin (Zhang et al., 2020). There is an increasing tendency to combine two fungicides with different mechanisms of action to increase activity and efficacy (Rashid et al., 2014; Cohen et al., 2018) and to delay the emergence of fungicide resistance (Dooley et al., 2015). There are some research reports that explore different classes of fungicides to identify compounds capable of inhibiting *Mucor* species growth and spore germination. This includes triazoles, benzimidazoles, strobilurins, and other chemical groups commonly used against fungal pathogens (Suárez-García et al., 2021; Yamleshwar and Rai, 2023; Bai et al., 2024).

Our results showed that difenoconazole has the strongest toxicity and the smallest EC_50_. The smaller the EC_50_, the stronger the toxicity of the agent and the better the antifungal effect (Halling-Sørensen, 2000; Hu et al., 2013). This fungicide belongs to the systemic fungicide, which can inhibit the formation of sporangium and prevent the infection of fungi. At the same time, it has a lasting protection and treatment effects, so as to improve crop yield and quality. Zeng et al. (2023) found that thiophanatemethyl, tebuconazole, and difenoconazole showed significant field control effects on pathogens of fruit brown rot on *Prunus salicina* var. *taoxingli*. The fungicide test results of our study broaden the control of brown rot disease on plum fruit and the selection of fungicides. Antifungal fungicides were tested for virulence *in vitro* only, with the EC_50_ value serving as a reference value during the *in vitro* test. Further verification is needed to screen out the effects in the field. Also, we need to examine the application method, the application time, the climate conditions, etc. The sensitivity of pathogenic fungi to fungicides may vary, so screening targeted fungicides is essential (Masiello et al., 2019). Mucor brown rot can be prevented and controlled with difenoconazole, followed by difenoconazole + azoxystrobin and mancozeb as alternatives or rotation agents, in order to prevent the development of pathogen resistance due to long-term or repeated use of the same fungicide.

## Data Availability

The datasets presented in this study can be found in online repositories. The names of the repository/repositories and accession number(s) can be found in the article/supplementary material.

## Footnotes

^1^ http://www.chinapesticide.org.cn/zwb/dataCenter
